# FOLFOX treatment response prediction in metastatic or recurrent colorectal cancer patients via machine learning algorithms

**DOI:** 10.1002/cam4.2786

**Published:** 2020-01-01

**Authors:** Wei Lu, Dongliang Fu, Xiangxing Kong, Zhiheng Huang, Maxwell Hwang, Yingshuang Zhu, Liubo Chen, Kai Jiang, Xinlin Li, Yihua Wu, Jun Li, Ying Yuan, Kefeng Ding

**Affiliations:** ^1^ Department of Colorectal Surgery The Second Affiliated Hospital Zhejiang University School of Medicine Hangzhou Zhejiang China; ^2^ Cancer Institute (Key Laboratory of Cancer Prevention and Intervention, China National Ministry of Education, Key Laboratory of Molecular Biology in Medical Sciences, Zhejiang Province, China) The Second Affiliated Hospital Zhejiang University School of Medicine Hangzhou Zhejiang China; ^3^ Department of Medical Oncology The Second Affiliated Hospital Zhejiang University School of Medicine Hangzhou Zhejiang China; ^4^ Department of Toxicology School of Public Health Zhejiang University Hangzhou Zhejiang China

**Keywords:** colorectal cancer, FOLFOX, machine learning algorithm, microarray meta‐analysis

## Abstract

Early identification of metastatic or recurrent colorectal cancer (CRC) patients who will be sensitive to FOLFOX (5‐FU, leucovorin and oxaliplatin) therapy is very important. We performed microarray meta‐analysis to identify differentially expressed genes (DEGs) between FOLFOX responders and nonresponders in metastatic or recurrent CRC patients, and found that the expression levels of WASHC4, HELZ, ERN1, RPS6KB1, and APPBP2 were downregulated, while the expression levels of IRF7, EML3, LYPLA2, DRAP1, RNH1, PKP3, TSPAN17, LSS, MLKL, PPP1R7, GCDH, C19ORF24, and CCDC124 were upregulated in FOLFOX responders compared with nonresponders. Subsequent functional annotation showed that DEGs were significantly enriched in autophagy, ErbB signaling pathway, mitophagy, endocytosis, FoxO signaling pathway, apoptosis, and antifolate resistance pathways. Based on those candidate genes, several machine learning algorithms were applied to the training set, then performances of models were assessed via the cross validation method. Candidate models with the best tuning parameters were applied to the test set and the final model showed satisfactory performance. In addition, we also reported that MLKL and CCDC124 gene expression were independent prognostic factors for metastatic CRC patients undergoing FOLFOX therapy.

## Graphical abstract

We performed the microarray meta‐analysis to identify common differentially expressed genes between FOLFOX responders and non‐responders in metastatic or recurrent colorectal cancer patients, and built prediction models with machine learning algorithms.

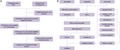

## 1 INTRODUCTION

Colorectal cancer (CRC) is still the third most commonly diagnosed cancer and the third leading cause of cancer‐related deaths.^1^, ^2^ Management of metastatic or recurrent CRC patients is a big challenge, since about 25% of CRC patients would present with metastatic lesions when firstly diagnosed, yet 50%‐60% of CRC patients finally developed metastatic lesions, with the majority of them being unresectable liver metastatic lesions.^3^, ^4^ Systemic therapies, including FOLFOX (5‐FU, leucovorin and oxaliplatin), FOLFIRI (5‐FU, leucovorin and irinotecan), FOLFOXIRI (5‐FU, leucovorin, oxaliplatin, and irinotecan), and CAPEOX (oxaliplatin and capecitabine), are the first‐line treatments for metastatic CRC patients.^5^, ^6^, ^7^ However, only about 50% of the CRC patients responded to first‐line systemic chemotherapies,^8^ while the remaining patients will suffer from delayed treatment and unnecessary side effects of antineoplastic drugs. Therefore, early identification of CRC patients who will probably be sensitive to a specific chemotherapy is very important.

Several studies have developed methods in various aspects to predict therapeutic responses of CRC patients toward some chemotherapies. For instance, Ahn et al reported that baseline CT texture could predict FOLFOX and FOLFIRI response in CRC patients with liver metastasis.^9^ In addition, DNA variations and specific gene expression profile in cancer tissues may have good predictive performance, since cancer genetic features are generally considered as one of the most important mechanisms leading to drug resistance.^10^, ^11^ Kap et al reported that several single nucleotide polymorphisms, which were involved in cellular metabolism and transport, could have potential predictive value for CRC patients undergoing oxaliplatin treatment.^12^ In addition, Kornmann et al conducted a randomized trial to show high mRNA level of thymidylate synthase in tumor biopsy samples was a valuable marker for predicting objective response during FOLFIRI treatment.^13^ However, FOLFOX treatment response prediction based on specific gene expression profile in metastatic or recurrent CRC patients is merely reported by few studies.^14^

Microarray and next‐generation sequencing could provide gene expression profiles and help identify differentially expressed genes (DEGs) between groups. High‐throughput gene expression profiles have been used to predict prognosis of CRC patients,^15^ identify stage II CRC patients who had high recurrence risk^16^ and predict patients' response toward certain chemotherapy.^17^ Nevertheless, it has been reported by a number of studies that results of microarray data were poor in reproducibility and were sensitive to perturbations of data.^18^, ^19^ Furthermore, microarray datasets generally used thousands of probes, while only a limited number of samples were tested, which will decrease the accuracy of model prediction. Fortunately, microarray meta‐analysis could solve the above issues by combining the results of several microarray datasets, detecting DEGs across datasets and evaluating their heterogeneities.^20^

In the current study, we performed microarray meta‐analysis to identify DEGs between FOLFOX responders and nonresponders in metastatic or recurrent CRC patients, and functional annotation of those DEGs was performed. We then adopted several machine learning algorithms to establish prediction models in the training set data, and assessed performances of models via the cross validation method. Candidate models were applied to the test set and the final model prediction performance was reported. In addition, we also explored whether those candidate genes could become prognostic markers for metastatic or recurrent CRC patients undergoing FOLFOX therapy.

## 2 MATERIALS AND METHODS

### 2.1 Datasets searching and screening

The datasets searching process was performed in the GEO database (http://www.ncbi.nlm.nih.gov/geo/) and the ArrayExpress database (http://www.ebi.ac.uk/arrayexpress/). The searching strategy was: (“colorectal cancer” OR “CRC”) AND (“FOLFOX”). In addition, published literatures were also manually retrieved in the PubMed database to avoid potentially missing datasets. The datasets searching process was conducted up to January 2018. Two independent researchers (Wei Lu and Dongliang Fu) screened the search results independently. First, duplicated datasets were removed, afterward, titles and brief descriptions of datasets were reviewed, and finally, datasets were assessed for eligibility according to the inclusion criteria: samples were primary or metastatic lesions of metastatic or recurrent CRCs; patients received first‐line FOLFOX regimen (patients received FOLFOX in combination with bevacizumab therapies were excluded), and all samples were obtained before FOLFOX regimen; expression microarrays were performed and raw data were available; FOLFOX treatment response statuses were available.

### 2.2 Data extraction and microarray data preprocessing

We extracted the following information of the included datasets: series accession number, microarray platform, year of data submission, source of specimens, number of specimens, gender, tissue type, regimen, response evaluation, and response rate. All lesions of each patient were assessed by image examination after FOLFOX therapy, and tumor response was evaluated according to RECIST recommendations.^21^ Responders represented complete response and partial response, while nonresponders represented stable disease and progressive disease. Raw data of each datasets were downloaded from the GEO database (http://ftp.ncbi.nih.gov/geo/series/). Raw data (CEL files) of each datasets were read into R and converted to the AffyBatch object using the ReadAffy function of the “affy” package (version 1.56.0) in R.^22^ Background correction, normalization, and summarization were performed using the MAS5 algorithm,^23^ then the log2 transformation was applied to the expression matrix. We used nsFilter function of the “genefilter” package (version 1.60.0) in R to filter multiple probe sets, which mapped to the same Entrez Gene ID, and removed noninformative probe sets according to the value of IQR (interquartile range).^24^ The expression matrix was then annotated by the “annotate” package (version 1.56.1) and the “hgu133plus2.db” package (version 3.2.3).^25^, ^26^ In addition, response statuses of patients were added according to the requirements of the “MetaDE” package (version 1.0.5) in R.^27^

### 2.3 Microarray meta‐analysis and differentially expressed genes identification

We then performed microarray meta‐analysis following the guidelines proposed by Ramasamy et al.^28^ First, we extracted the common genes across multiple studies and sorted them in the same order. Then we used the “MetaQC” package (version 0.1.13) in R to implement the objective quality control, including IQC (internal quality control index), EQC (external quality control index), CQCg (consistency of differential expression quality control in genes), CQCp (consistency of differential expression quality control in pathways), AQCg (accuracy of differential expression quality control in genes), and AQCp (accuracy of differential expression quality control in pathways).^29^ DEGs were identified using the “MetaDE” package (version 1.0.5) in R according to the FOLFOX response status,^27^, ^30^ then heterogeneity was evaluated using Q statistics and *P* value for Q statistics more than .05 indicated no significant heterogeneity existed across studies. The moderated t statistic was used to calculate the effect size of each gene based on the robust permutation inferences (number of permutations = 300). Effect sizes were pooled via the random‐effect model method,^31^ and the false discovery rate (FDR) controlling was carried out by the Benjamini‐Hochberg procedure, with FDR cutoff value of 0.3 to select candidate genes for further machine learning practice.^32^ We also displayed a heat map to visualize DEGs across studies, and we used the correlation plot to visualize correlation coefficients between variables.

### 2.4 Kyoto Encyclopedia of Genes and Genomes (KEGG) pathway term enrichment analysis and gene ontology (GO) term enrichment analysis

Kyoto Encyclopedia of Genes and Genomes (KEGG) pathway term enrichment was performed using the Metascape online tool (http://metascape.org).^33^ For each DEG (*P* < .05 in the MetaDE results), we first identified statistically enriched KEGG pathway terms, *P* values for hypergeometric distribution and enrichment factors (the ratio between observed gene counts and the gene counts expected by chance). Enrichment background was set as all genes in the genome. Significant KEGG pathway terms were hierarchically clustered based on κ‐statistical similarities among gene memberships, and 0.3 was set as the κ cutoff value for clustering. In addition, a subset of representative KEGG pathway terms from these clusters were converted to a network.

Gene ontology (GO) enrichment of DEGs in cellular component ontology and biological process ontology was performed by the “clusterProfiler” package in R.^34^ Enrichment background was set as all genes in the genome, and the *P* cutoff value after Benjamini‐Hochberg adjustment was set as .05.^32^ Cluster network and the tree of GO terms were also displayed for visualization.

### 2.5 Screening and cross validation of machine learning models

We chose the dataset http://www.ncbi.nlm.nih.gov/geo/query/acc.cgi?acc=GSE28702, which had the largest sample size among all included studies, to apply machine learning algorithms. First, we extracted the expression matrix from the dataset, which was composed of expression value of candidate genes, sample ID, and FOLFOX treatment response status. Data preprocess was performed via the “preprocess” function of the “caret” package (version 6.0‐77) in R, and we adopted the “center” and “scale” methods.^35^ Next, samples were randomly assigned to training set (60%) and test set (40%) using the “sample” function in R. Then we used fivefold cross validation (“createFolds” function of the “caret” package) for 20 random replications in the training set to evaluate model performance.^35^, ^36^ A total of six machine learning algorithms were trained using relevant R packages: k‐nearest neighbor (KNN) of the “class” package,^37^ support vector machine (SVM) of the “e1071” package (version 1.6‐8),^38^ gradient boosting machine (GBM) of the “gbm” package (version 2.1.3),^39^ decision tree of the “tree” package (version 1.0‐37),^40^ random forest of the “randomForest” package (version 4.6‐12),^41^ and neural network of the “RSNNS” package (version 0.4‐9).^42^ The impact of various tuning parameters on model performance was also evaluated in the cross validation procedure, and the best tuning parameters were selected to represent the performance of the machine learning algorithms, respectively. Assessments of model performance were mainly consisted of accuracy, sensitivity, specificity, and Youden index. Finally, the top 3 machine learning algorithms, with their own best tuning parameters, were applied to the test set to predict FOLFOX treatment response.

### 2.6 Statistical analysis

We used R (version 3.4.2), SPSS 22, and GraphPad Prism 6 to perform data process and statistical analyses. Microarray data preprocessing, microarray meta‐analysis, enrichment analysis, and machine learning algorithms were described in the previous sections. Comparisons among several machine learning algorithms were performed using ANOVA. ROC (receiver operating characteristic) curves were plotted and AUC (area under the curve) was calculated using the “pROC” package (1.12.1),^43^ and AUC was compared with 0.5 using the *Z*‐test, which represented results of random predictions. Survival analyses were performed in SPSS 22 and univariate Cox regression was used to screen variables using the “Enter” method under a *P* value of .05. Variables which had significant prognostic values in the univariate Cox regression were included in the final multivariate Cox regression. A *P* value less than .05 showed statistical significance unless otherwise stated.

## 3 RESULTS

### 3.1 Characteristics of the included datasets

**Figure 1:**
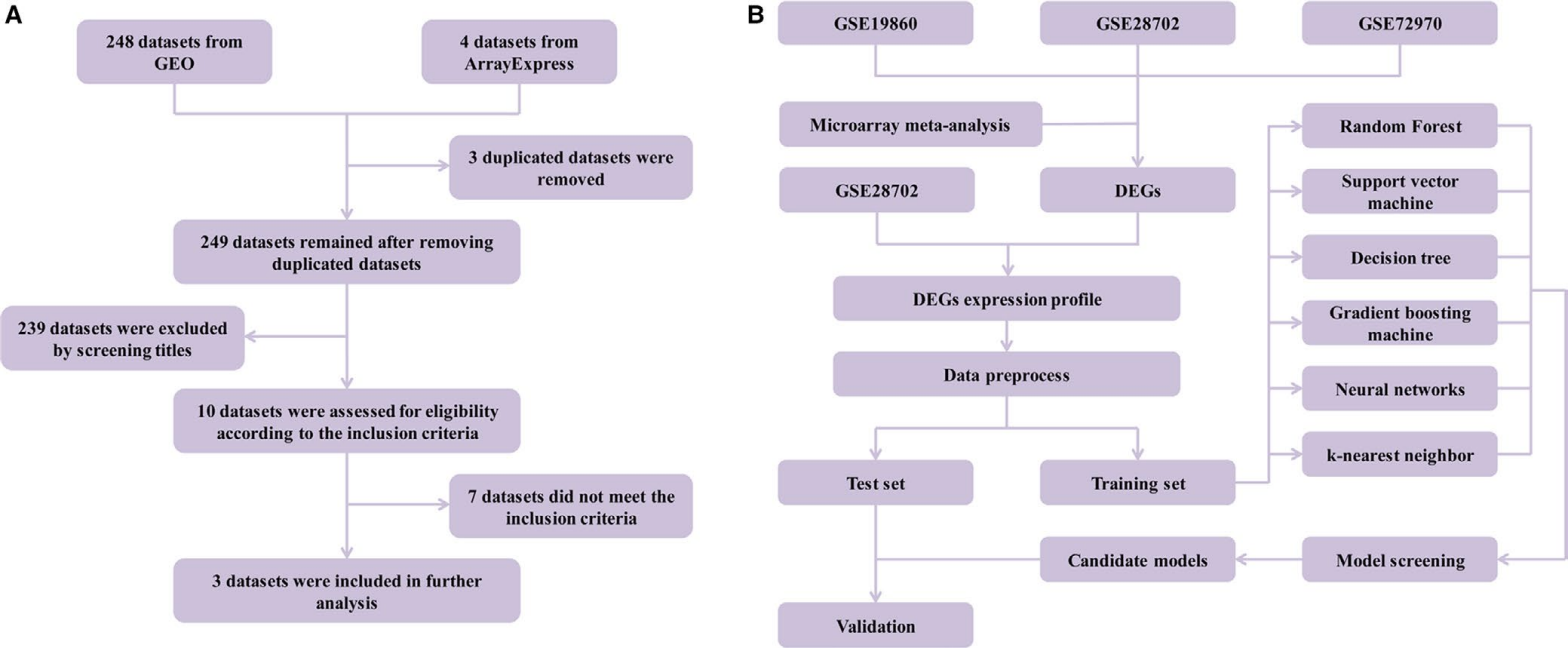
A, Flow diagram of datasets screening and selection. B, Flow diagram of identifying differentially expressed genes and building models via machine learning algorithms

We exported 248 datasets and four datasets when searching the GEO database and the ArrayExpress database, respectively (Figure 1A), then three duplicated datasets were removed. After screening titles and summaries of 249 datasets, 10 datasets remained and were assessed for eligibility according to the inclusion criteria. Finally, three datasets were included in the further analysis and characteristics of datasets were displayed in Table 1. In brief, all the datasets used the same microarray platform (Affymetrix Human Genome U133 Plus 2.0 Array). http://www.ncbi.nlm.nih.gov/geo/query/acc.cgi?acc=GSE19860 had 29 metastatic or recurrent CRCs, while http://www.ncbi.nlm.nih.gov/geo/query/acc.cgi?acc=GSE28702 and http://www.ncbi.nlm.nih.gov/geo/query/acc.cgi?acc=GSE72970 were composed of 83 and 32 metastatic CRCs, respectively. Tissue types of http://www.ncbi.nlm.nih.gov/geo/query/acc.cgi?acc=GSE19860 and http://www.ncbi.nlm.nih.gov/geo/query/acc.cgi?acc=GSE72970 were all primary lesions, and http://www.ncbi.nlm.nih.gov/geo/query/acc.cgi?acc=GSE28702 had 56 primary lesions and 23 metastatic lesions to the liver, three metastatic lesions to the peritoneum, and one metastatic lesion to the lung. Treatment response evaluation was performed at the end of the first‐line FOLFOX treatment, and response rates varied from 31.03% to 60.60% across datasets.

**Table 1:** Characteristics of the included datasets

Series accession	Platforms	Year of submission	Specimens source	Number of specimens	Male/Female	Tissue type	Regimen	Response evaluation	Response rate
http://www.ncbi.nlm.nih.gov/geo/query/acc.cgi?acc=GSE19860	Affymetrix Human Genome U133 Plus 2.0 Array	2010	Department of Surgical Oncology, University of Tokyo	29	NA	Primary colorectal cancers	mFOLFOX6	The best observed response at the end of the first‐line treatment	31.03%
http://www.ncbi.nlm.nih.gov/geo/query/acc.cgi?acc=GSE28702	Affymetrix Human Genome U133 Plus 2.0 Array	2011	Teikyo University Hospital and Gifu University Hospital	83	54/29	56 primary colorectal cancers, 23 metastatic lesions to the liver, 3 metastatic lesions to the peritoneum, and 1 metastatic lesion to the lung	mFOLFOX6	Assessment by computed tomography after four cycles of mFOLFOX6 therapy	50.60%
http://www.ncbi.nlm.nih.gov/geo/query/acc.cgi?acc=GSE72970	Affymetrix Human Genome U133 Plus 2.0 Array	2015	REGP, COSIVAL and BIOCOLON cohorts	32	19/13	Primary colorectal cancers	FOLFOX	The best observed response of first‐line treatment	60.60%

Note

mFOLFOX6: modified FOLFOX6

### 3.2 Microarray meta‐analysis and differentially expressed genes identification

The microarray meta‐analysis and machine learning work flow were presented in Figure 1B. First, we performed data preprocess in each datasets and extracted expression matrix accordingly. Then we calculated six quality control indicators in each datasets, as shown in Table S1. All datasets had similar ranks and performed well in terms of IQC and EQC, suggesting that all datasets had good internal and external homogeneity. Nevertheless, low CQCg, AQCg, CQCp, and AQCp score implied that DEGs were inconsistent across datasets, thus we believed that DEGs identification through microarray meta‐analysis was necessary and it could help identify FOLFOX treatment responders in metastatic or recurrent CRC patients.

Next, we analyzed DEGs between FOLFOX nonresponders and responders by performing the moderated *t* test to calculate the effect size of each gene, then the random‐effect model was utilized to pool results across datasets. We identified 778 DEGs using a *P* cutoff value of .05 (data not shown), and they were used to perform KEGG enrichment analysis and GO enrichment analysis.

### 3.3 KEGG enrichment analysis and GO enrichment analysis

**Figure 2:**
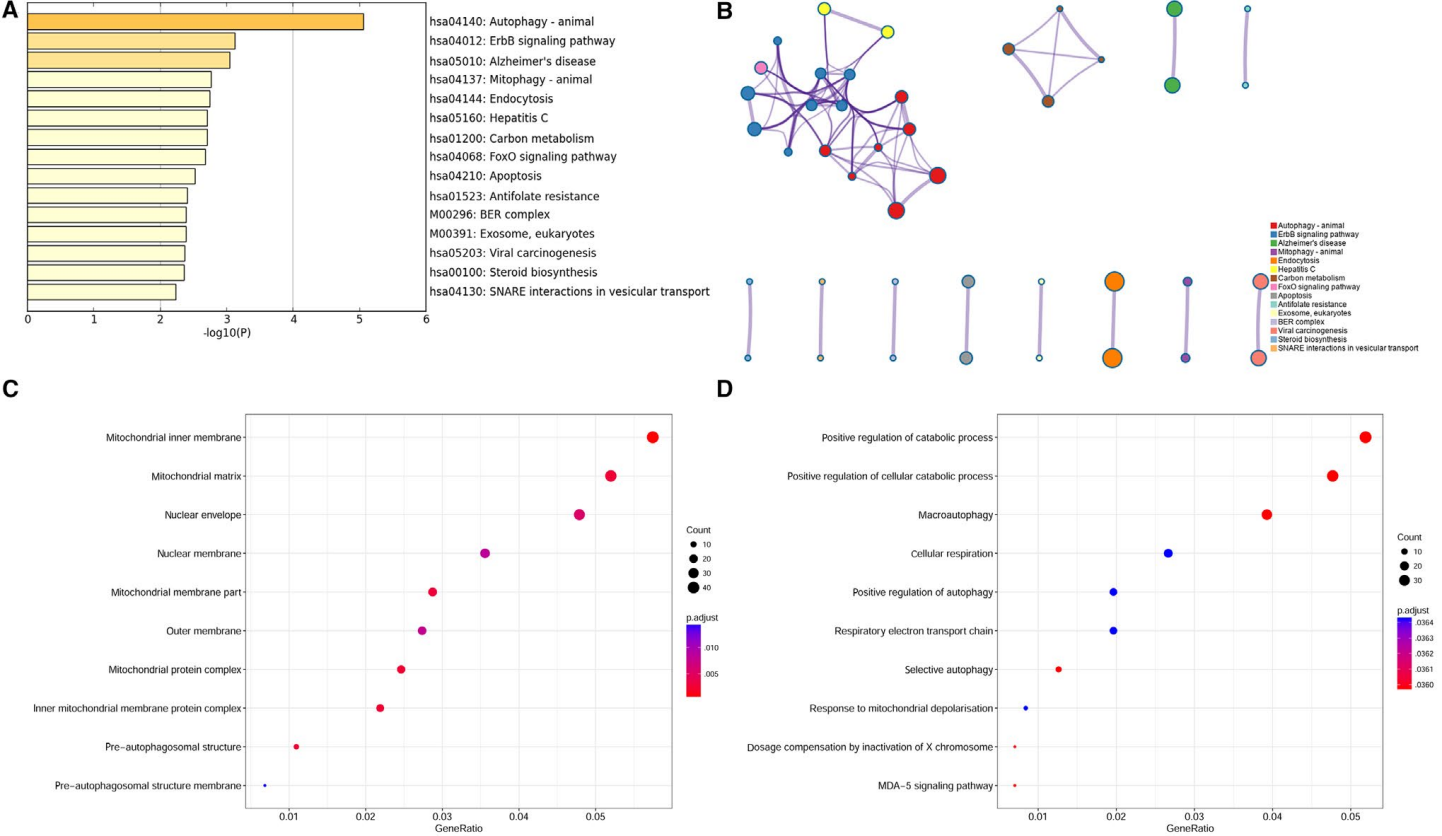
A, Bar plot of enriched KEGG terms based on differentially expressed genes. Darker colors indicated smaller *P* values. B, Network of enriched KEGG terms was colored by cluster ID. C, Dot plot of GO enrichment of differentially expressed genes in cellular component ontology. D, Dot plot of GO enrichment of differentially expressed genes in biological process ontology

We listed top 15 KEGG pathway terms in the KEGG enrichment analysis (Table S2 and Figure 2A), and we found those DEGs were significantly enriched in autophagy, ErbB signaling pathway, mitophagy, endocytosis, FoxO signaling pathway, apoptosis, antifolate resistance, etc In addition, autophagy and ErbB signaling pathway clusters were closely correlated (Figure 2B).

GO cellular component ontology enrichment analysis showed that those DEGs were significantly enriched in membrane structures and mitochondrial components, including mitochondrial inner membrane, mitochondrial matrix, mitochondrial protein complex, nuclear membrane, outer membrane, and preautophagosomal structure membrane (Figure 2C and S1A). Besides, GO biological process ontology enrichment analysis showed significant enrichment in positive regulation of catabolic process, macroautophagy, cellular respiration, and response to mitochondrial depolarization (Figure 2D and S1B), which was in consistent with the results of KEGG enrichment analysis.

### 3.4 FOLFOX treatment response prediction via machine learning algorithms

**Figure 3:**
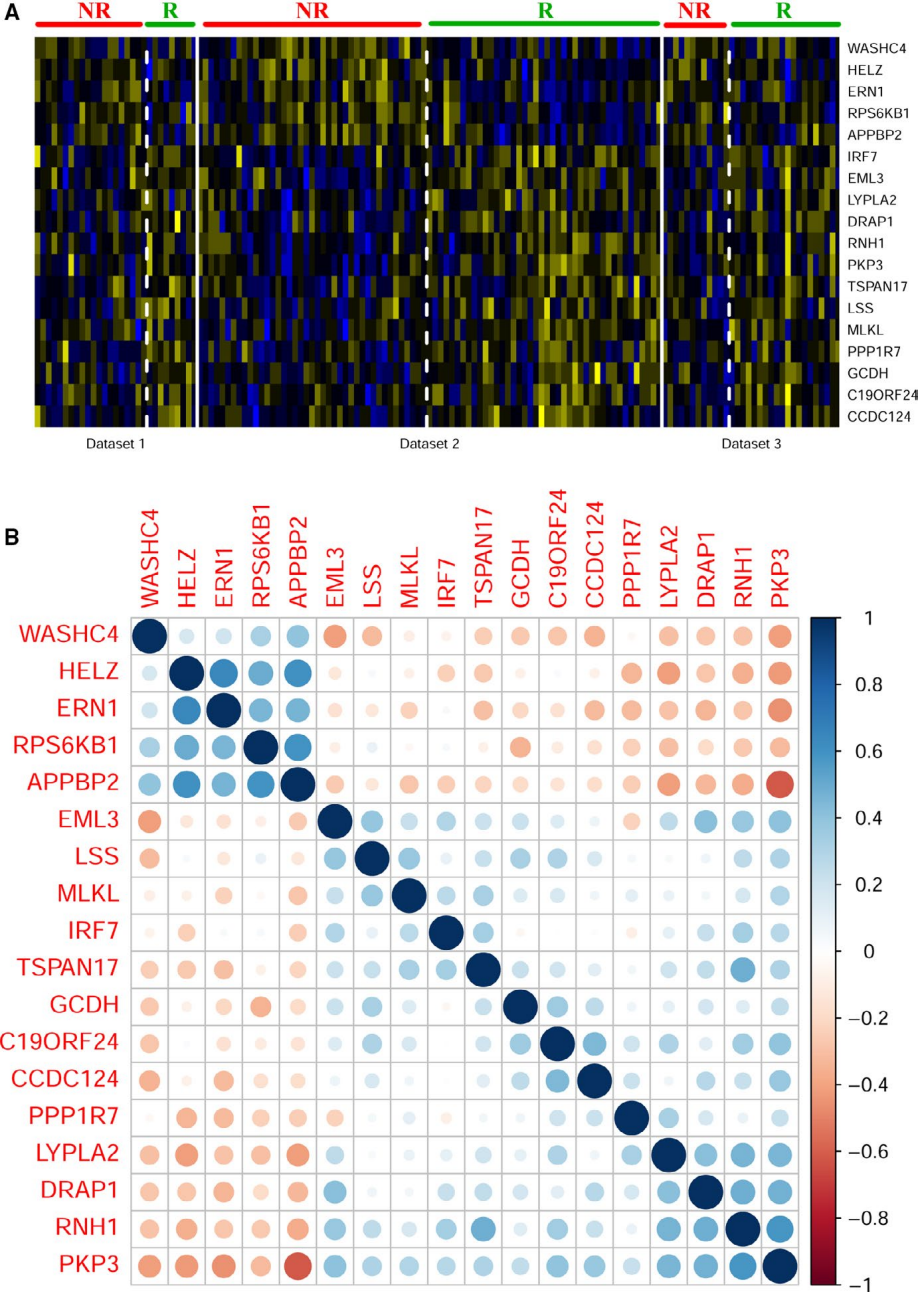
A Heat map of 18 differentially expressed genes used in prediction models. Blue color indicated gene downregulation and yellow color indicated gene upregulation. NR indicates nonresponders and R indicates responders. Dataset 1, dataset 2, and dataset 3 are http://www.ncbi.nlm.nih.gov/geo/query/acc.cgi?acc=GSE19860, http://www.ncbi.nlm.nih.gov/geo/query/acc.cgi?acc=GSE28702, and http://www.ncbi.nlm.nih.gov/geo/query/acc.cgi?acc=GSE72970, respectively. B Correlation coefficient plot of 18 differentially expressed genes in http://www.ncbi.nlm.nih.gov/geo/query/acc.cgi?acc=GSE28702. Blue color indicated positive correlation and red color indicated negative correlation

Significant gene numbers under various FDR cutoff values in microarray meta‐analysis were displayed in Figure S2, and top 18 candidate genes were selected for further machine learning practice under the FDR cutoff value of 0.3 (Table S3). No significant heterogeneity was observed according to the *P* value for Q statistics. As displayed in the heat map (Figure 3A), expression levels of five genes were downregulated in FOLFOX responders (WASHC4, HELZ, ERN1, RPS6KB1, and APPBP2), while expression levels of 13 genes were upregulated in FOLFOX responders (IRF7, EML3, LYPLA2, DRAP1, RNH1, PKP3, TSPAN17, LSS, MLKL, PPP1R7, GCDH, C19ORF24, and CCDC124), with the logFC value varying from −0.751 to 0.621.

The dataset http://www.ncbi.nlm.nih.gov/geo/query/acc.cgi?acc=GSE28702, which had the largest sample size, was chosen to apply machine learning algorithms. The expression matrix of 18 candidate genes was extracted, and the correlation coefficient plot showed that downregulated and upregulated genes in FOLFOX responders were mainly positively correlated within their own group, yet negatively correlated with genes from another group (Figure 3B). No correlation coefficients were more than 0.7, thus no genes were filtered and 18 candidate genes were all included in the further analysis. Data preprocess of the expression matrix was performed using the “center” and “scale” methods via the “preprocess” function of the “caret” package (version 6.0‐77).

Next, samples in http://www.ncbi.nlm.nih.gov/geo/query/acc.cgi?acc=GSE28702 were randomly assigned to the training set (60%) and the test set (40%). We used fivefold cross validation method for 20 random replications in the training set to evaluate model performance. Six machine learning algorithms were tested, including KNN, SVM, GBM, decision tree, random forest, and neural network. The impact of various tuning parameters on model performance was also evaluated in the cross validation procedure, and the best tuning parameters were selected to represent the performance of the corresponding machine learning algorithm. Those tuning parameters were *k* in KNN; Gamma, cost, and kernel type in SVM; the total number of trees to fit and shrinkage in GBM; the number of trees to grow and the number of variables randomly sampled as candidates at each split in random forest; the number of units in the hidden layers and the learning function type in neural network.

**Figure 4:**
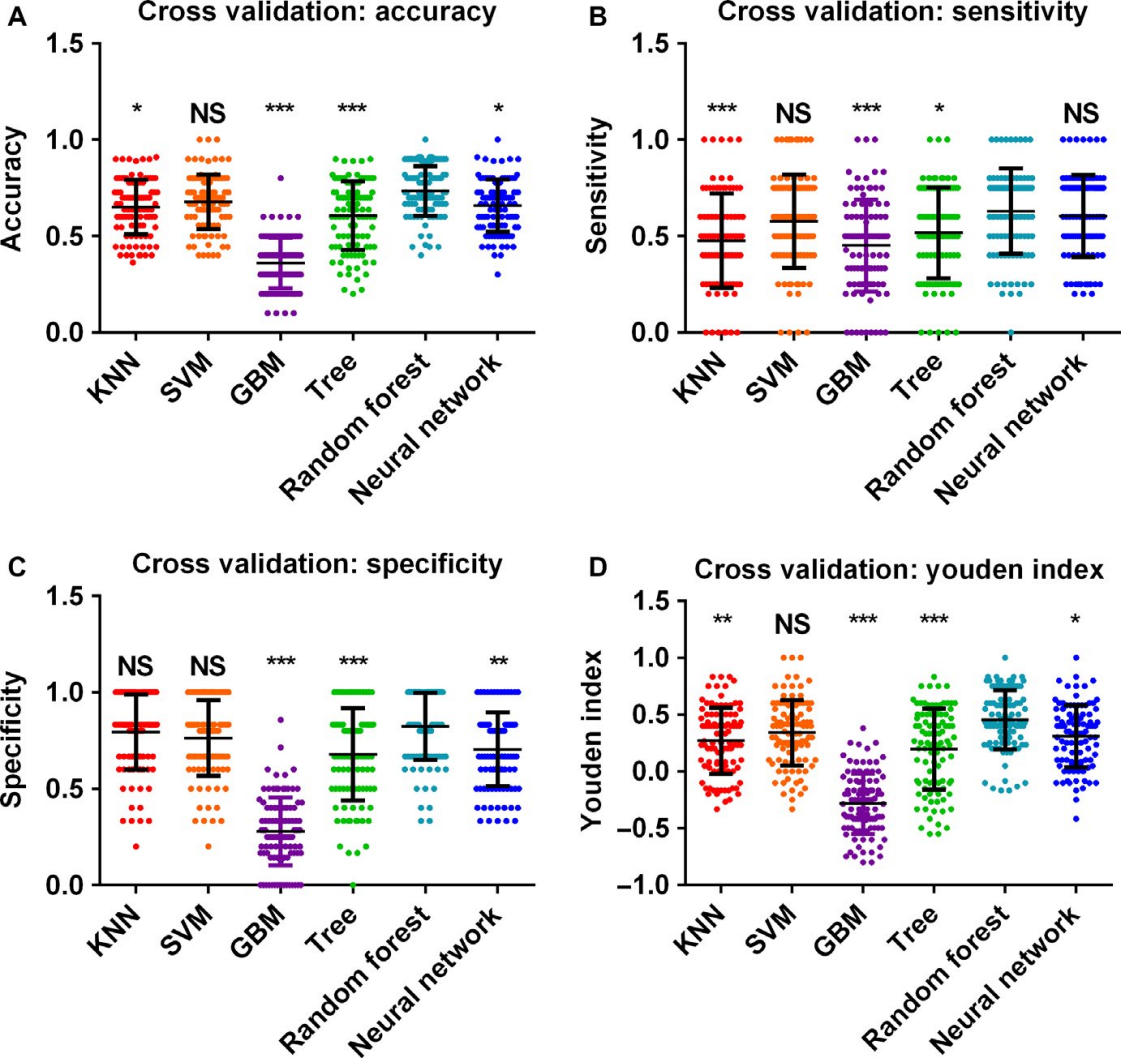
Cross validation results using KNN, SVM, GBM, tree, random forest, and neural network algorithms. Model performances were determined according to prediction (A) accuracy, (B) sensitivity, (C) specificity, and (D) Youden index, each dot represented onefold of cross validation result. Results were compared by Kruskal‐Wallis ANOVA analysis, and multiple comparisons are performed by Dunn's tests. Significances were labeled between the random forest algorithm and other algorithms. *P < .05, **P < .01, ***P < .005

Then assessments of model performance were performed in cross validation sets according to accuracy, sensitivity, specificity, and Youden index (Figure 4 and Table S4). The top 3 machine learning algorithms were random forest, SVM, and neural network algorithms. There was no significant difference between the SVM and the random forest algorithm in terms of all statistics; however, the neural network algorithm was significantly inferior to the random forest algorithm in terms of accuracy, specificity, and Youden index. Afterward, we applied these top 3 machine learning algorithms in the test set to predict FOLFOX response, and the prediction results were displayed in Figure S3. SVM, random forest, and neural network algorithms had an AUC of 0.827 (95% CI: 0.670‐0.984, *P* < .01), 0.877 (95% CI: 0.747‐1.00, *P* < .01), and 0.800 (95% CI: 0.638‐0.962, *P* < .01) accordingly (Figure S4). Using the dichotomize scores as prediction results, as shown in Table 2, the SVM algorithm ranked first with a sensitivity of 0.900 (95% CI: 0.669‐0.982) and a specificity of 0.692 (95% CI: 0.389‐0.896). The random forest algorithm was comparable to the SVM algorithm with a sensitivity of 0.850 (95% CI: 0.611‐0.960) and the same specificity. However, the neural network algorithm ranked last with a sensitivity of 0.800 (95% CI: 0.557‐0.934) and a relatively low specificity of 0.538 (95% CI: 0.261‐0.796). In addition, we also calculated the positive likelihood ratio (PLR) and the negative likelihood ratio (NLR) of each algorithm. The SVM algorithm had a PLR of 2.925 (95% CI: 1.278‐6.697) and a NLR of 0.144 (95% CI: 0.036‐0.575), while the random forest algorithm had similar results. However, the neural network algorithm ranked last, with a PLR of 1.733 (95% CI: 0.926‐3.244) and a NLR of 0.371 (95% CI: 0.136‐1.015).

**Table 2:** Model prediction results in the test set

	SVM	Random forest	Neural network
Sensitivity	0.900	0.850	0.800
95% CI	(0.669‐0.982)	(0.611‐0.960)	(0.557‐0.934)
Specificity	0.692	0.692	0.538
95% CI	(0.389‐0.896)	(0.389‐0.896)	(0.261‐0.796)
PLR	2.925	2.762	1.733
95% CI	(1.278‐6.697)	(1.197‐6.373)	(0.926‐3.244)
NLR	0.144	0.217	0.371
95% CI	(0.036‐0.575)	(0.071‐0.658)	(0.136‐1.015)

Abbreviations: PLR, positive likelihood ratio; NLR, negative likelihood ratio; SVM, support vector machine.

### 3.5 Identifying prognostic genes on overall survival for metastatic CRC patients undergoing FOLFOX therapy

After confirming the predictive value of these machine learning algorithms, we then investigated whether these candidate genes could be prognostic factors of survival as well. First, we performed the univariate Cox regression in http://www.ncbi.nlm.nih.gov/geo/query/acc.cgi?acc=GSE28702 (Table S5). We found that PKP3, LSS, MLKL, C19ORF24, and CCDC124, which were all upregulated genes in FOLFOX responders, were statistically significant positive prognostic factors on overall survival. However, we did not find statistically significant negative prognostic factors among 18 genes. In the multivariate Cox regression, we found that only MLKL (HR = 0.358, 95% CI: 0.178‐0.717, *P* = .004) and CCDC124 (HR = 0.563, 95% CI: 0.336‐0.943, *P* = .029) genes indicated improved overall survival significantly.

## 4 DISCUSSIONS

FOLFOX is one of the most frequently used first‐line chemotherapy regimens for metastatic CRC patients, yet only about 50% of the CRC patients had objective responses after FOLFOX treatment.^8^ Unresponsive patients toward first‐line therapy usually suffered from progressive diseases, unnecessary but serious side effects of antineoplastic medications, and massive economic burdens. Therefore it is of great importance to identify CRC patients who will be sensitive to a specific chemotherapy regimen. Researchers have reported methods in various aspects to predict therapeutic responses of CRC patients toward some chemotherapies,^9^, ^12^, ^13^ while FOLFOX treatment response prediction in metastatic or recurrent CRC patients is merely reported by few studies.^14^

In the current study, we performed the microarray meta‐analysis to identify common DEGs between FOLFOX responders and nonresponders in metastatic or recurrent CRC patients, and we found those DEGs were significantly enriched in autophagy, ErbB signaling pathway, mitophagy, endocytosis, FoxO signaling pathway, apoptosis, antifolate resistance, etc Consistent with previous studies, autophagy has been supposed to act as defensive mechanisms against Oxaliplatin in CRC^44^ and mitophagy inhibition was reported to enhance anticancer drug sensitivity in a variety of cancers,^45^ suggesting autophagy and mitophagy may be a promising therapeutic target for CRC patients undergoing FOLFOX therapy.

Machine learning algorithms were predominant approaches that could build predictive models based on microarray data. Using the top 18 gene panel, we applied several machine learning algorithms to predict FOLFOX response. After cross validation in the training set, random forest, SVM, and neural network algorithms were applied to the test set. We found both the SVM and random forest algorithms ranked first with a high sensitivity and a moderate specificity, yet the neural network algorithm was inferior to the above two algorithms. In addition, our results were in accordance with previous reports that SVM and random forest algorithms were the most accurate algorithms in the aspect of microarray‐based classification.^46^ Among the top 18 gene panel, we found that high expression of MLKL and CCDC124, which were upregulated genes in FOLFOX responders, indicated significantly improved overall survival in metastatic CRC patients undergoing FOLFOX treatment. Although CCDC124 has not been reported to be associated with antineoplastic drug resistance, MLKL is a pseudokinase that plays a pivotal role in tumor necrosis factor‐induced necroptosis and mediates the antimicrobial peptide HPA3P‐induced necrotic death in colon cancer.^47^ Moreover, Sun et al have developed a nanoscale cationic liposome system encapsulating MLKL‐pDNA, SMAC mimetic, and zVAD to solve the multidrug resistance in colon cancer cells.^48^ These previous researches and our findings implied that MLKL could be a potential therapeutic target for FOLFOX‐resistant metastatic CRC patients.

In addition, FOLFOX and FOLFIRI therapies share two chemotherapeutic medications and they differ in one single agent, and FOLFIRI therapy is also widely used in metastatic or recurrent CRC patients. We also have tried to apply the top 3 machine learning algorithms (random forest, SVM, and neural network) to a FOLFIRI dataset (http://www.ncbi.nlm.nih.gov/geo/query/acc.cgi?acc=GSE62080), using the same pipeline as FOLFOX response prediction before. We found SVM, random forest, and neural network algorithms had an AUC of 0.676 (95% CI: 0.438‐0.914, *P* = .147), 0.667 (95% CI: 0.426‐0.908, *P* = .173), and 0.778 (95% CI: 0.576‐0.979, *P* < .01) accordingly. Since the best FOLFOX prediction algorithms were the random forest algorithm and SVM algorithm, but their performances dropped greatly when predicting FOLFIRI response, therefore, we believed that these two predictors were specifically trained for predicting FOLFOX response. However, it was interesting to find that the neural network algorithm had moderate performance when predicting FOLFIRI response, which may be due to the effect of two overlapped chemotherapeutic medications between FOLFOX and FOLFIRI therapies.

It is worth noting that our study had a number of strengths. First, CRC patients in our study received first‐line FOLFOX treatment, and the influence of other chemotherapies and target agents such as bevacizumab was eliminated. Second, microarray data were poor in reproducibility and were sensitive to perturbations of data, therefore DEGs across datasets or platforms may be inconsistent.^18^, ^19^ Fortunately, microarray meta‐analysis could help us solve this issue by combining the results of several microarray datasets,^20^ and our results were more reliable and universal than results from single microarray dataset. Third, we tested six machine leaning algorithms and the model performances were reflected by the cross validation results. In addition to FOLFOX response prediction, we also identified prognostic genes on overall survival for metastatic CRC patients undergoing FOLFOX therapy.

However, our study was limited in some aspects as well. For instance, FOLFOX treatment response prediction and survival analysis were only performed in http://www.ncbi.nlm.nih.gov/geo/query/acc.cgi?acc=GSE28702 and another two datasets were not included in the analysis due to their small sample sizes. Besides, http://www.ncbi.nlm.nih.gov/geo/query/acc.cgi?acc=GSE28702 contained both primary CRC samples and metastatic CRC samples, but we did not perform further subgroup analysis due to the limited sample size and sample type, despite the fact that metastatic lesions may have significant differences from primary lesions. For metachronous metastatic cancers, primary lesions may also differ from metastatic lesions if long time intervals exist between the onset of primary tumors and metastatic lesions. Fortunately, the model prediction performance was satisfactory to some extent, and we believed our model prediction performance will be improved if we build models using primary CRC samples and metastatic CRC samples separately. Metastatic CRC patients are recommended to use chemotherapies plus target agents unless contraindicated,^49^ but whether adding a target agent will influence the prediction results was unclear. Besides, the microarray platform used in the study did not detect microRNA or long noncoding RNA, and those noncoding RNAs have been proved to play crucial roles in various biological processes, including oxaliplatin‐induced chemoresistance in CRC.^50^, ^51^ We believed our prediction model will perform better if those features were added.

In summary, we reported that WASHC4, HELZ, ERN1, RPS6KB1, and APPBP2 were downregulated, while IRF7, EML3, LYPLA2, DRAP1, RNH1, PKP3, TSPAN17, LSS, MLKL, PPP1R7, GCDH, C19ORF24, and CCDC124 were upregulated in FOLFOX responders compared with nonresponders in metastatic or recurrent CRC patients, and those genes could be potential therapeutic targets for FOLFOX‐resistant metastatic CRC. DEGs were significantly enriched in autophagy, ErbB signaling pathway, mitophagy, endocytosis, FoxO signaling pathway, apoptosis, and antifolate resistance pathways. In addition, SVM and random forest algorithms based on those DEGs could help predict FOLFOX response, meanwhile expression levels of MLKL and CCDC124 were independent prognostic factors for metastatic CRC patients undergoing FOLFOX therapy.

## CONFLICT OF INTERESTS

The authors have declared no conflict of interest.

## AUTHOR CONTRIBUTION

Kefeng Ding had the right to grant on behalf of all authors. Wei Lu contributed to the conception and design of the study. Wei Lu and Dongliang Fu contributed to database search, data process, and statistical analysis. Xiangxing Kong, Zhiheng Huang, Maxwell Hwang, Kai Jiang, Yihua Wu, Jun Li, and Ying Yuan gave valuable suggestions about the project. Wei Lu, Yingshuang Zhu, Liubo Chen, and Xinlin Li drafted the manuscript. All authors have commented on the manuscript and approved the final draft.
