# Supplementary figures and images for: FOLFOX treatment response prediction in metastatic or recurrent colorectal cancer patients via machine learning algorithms
Source: Cancer Med. 2020 Jan 1;9(4):1419–29. doi: 10.1002/cam4.2786 (PMC7013065; doi:10.1002/cam4.2786)

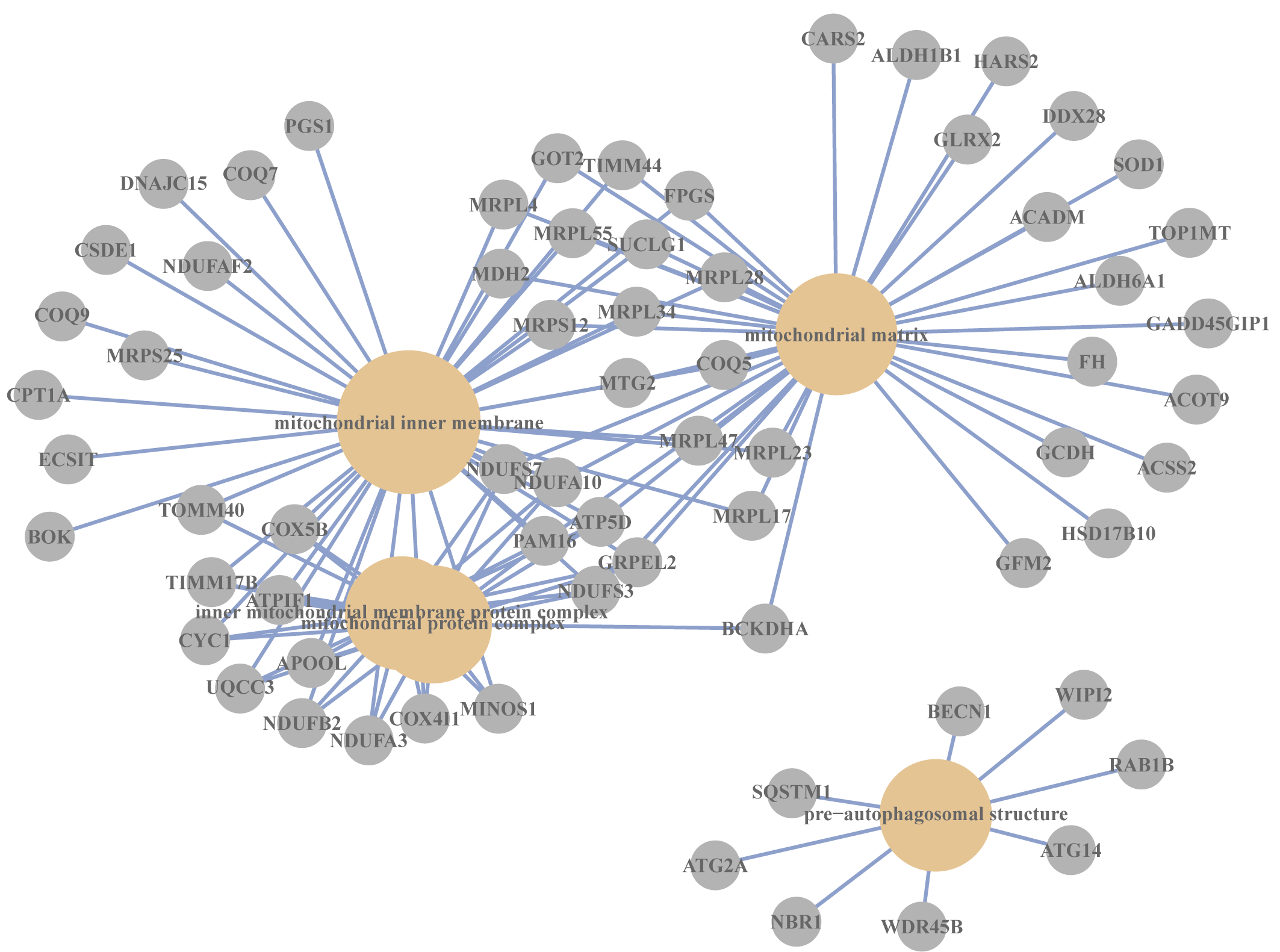

Supplement: Supplementary file 1 [file CAM4-9-1419-s001.tif]

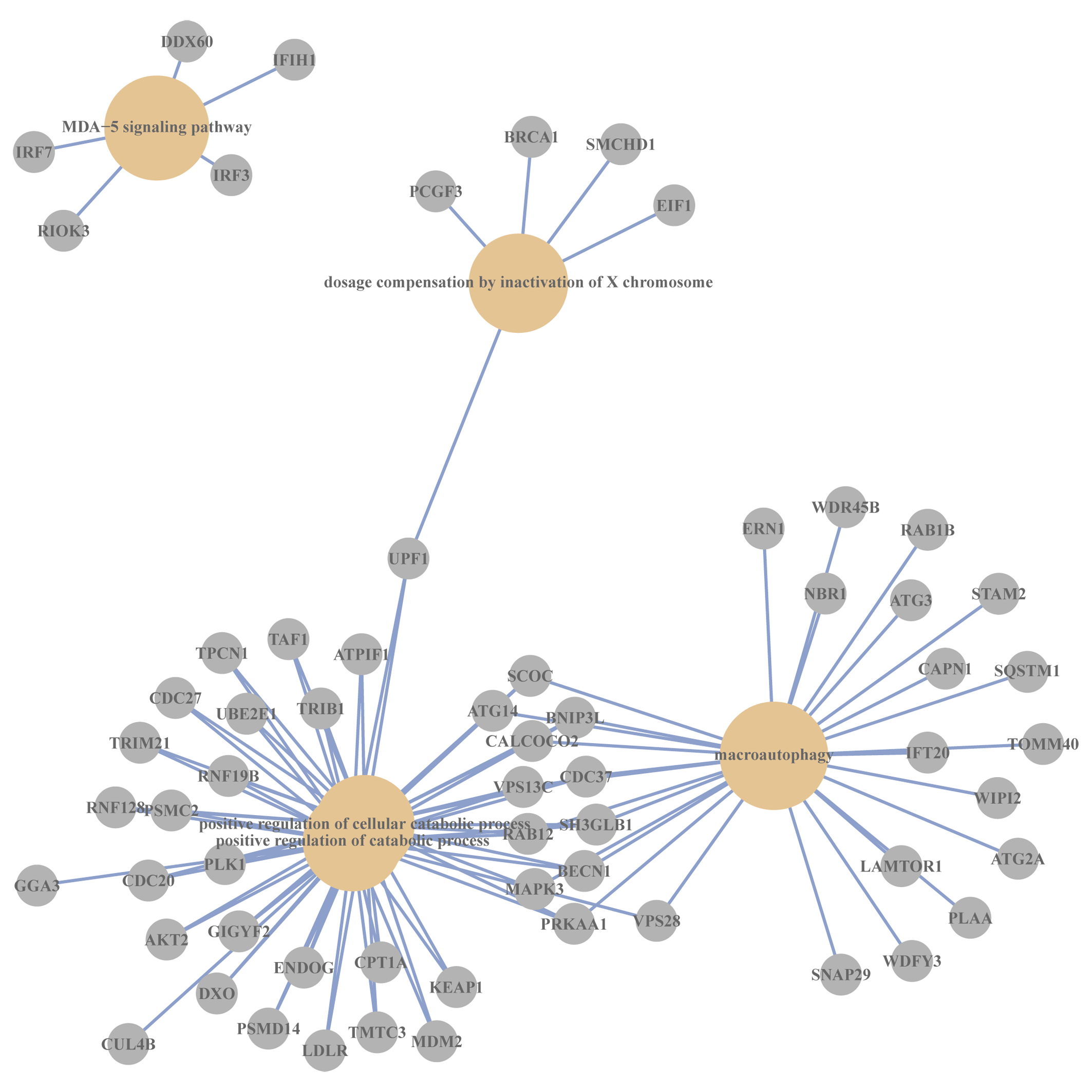

Supplement: Supplementary file 2 [file CAM4-9-1419-s002.tif]

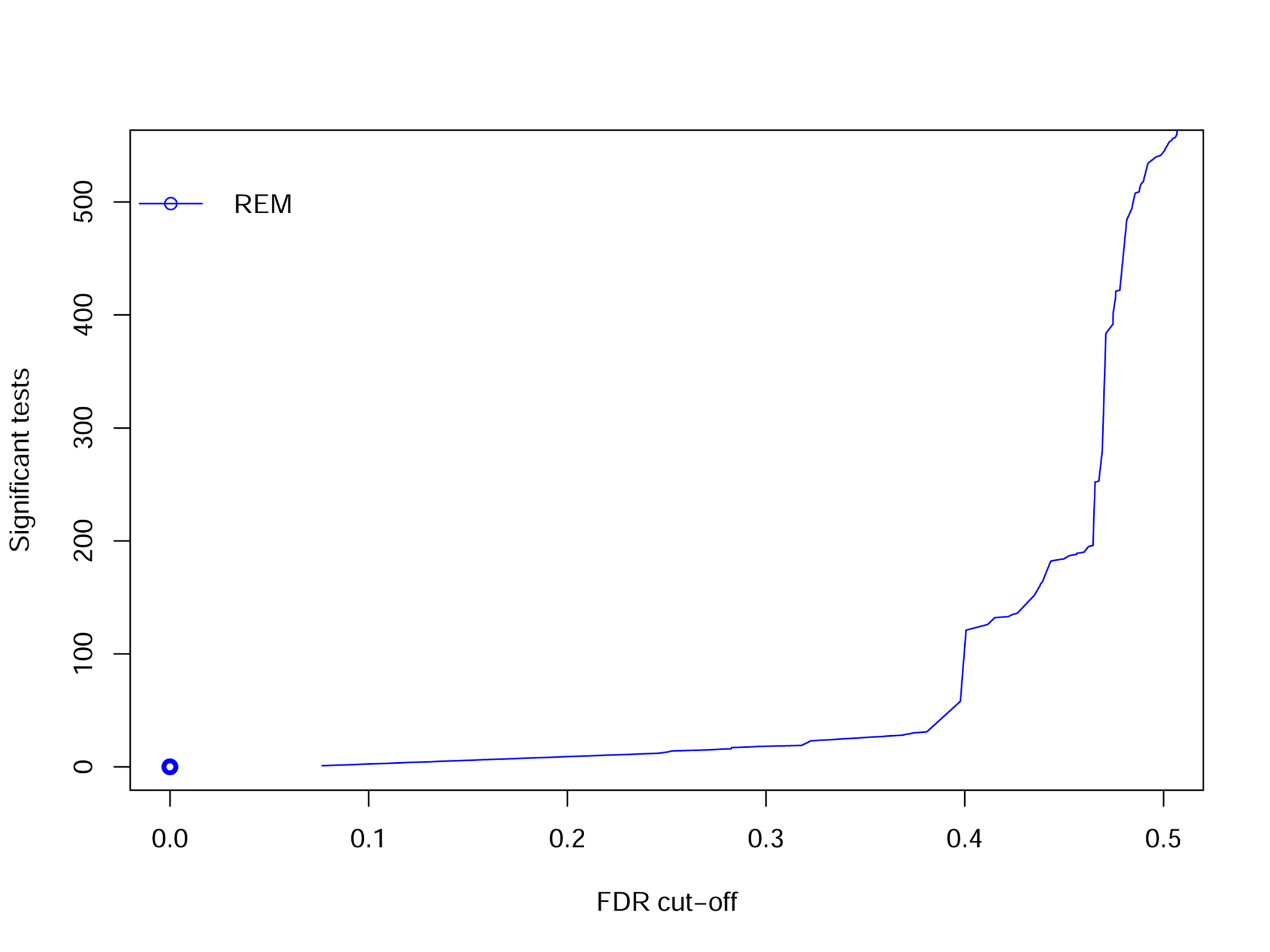

Supplement: Supplementary file 3 [file CAM4-9-1419-s003.tif]

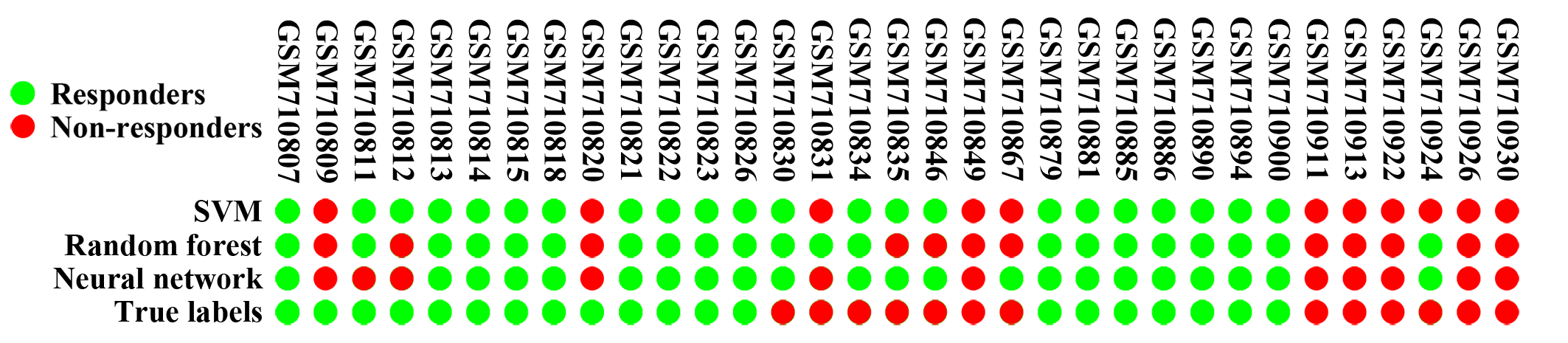

Supplement: Supplementary file 4 [file CAM4-9-1419-s004.tif]

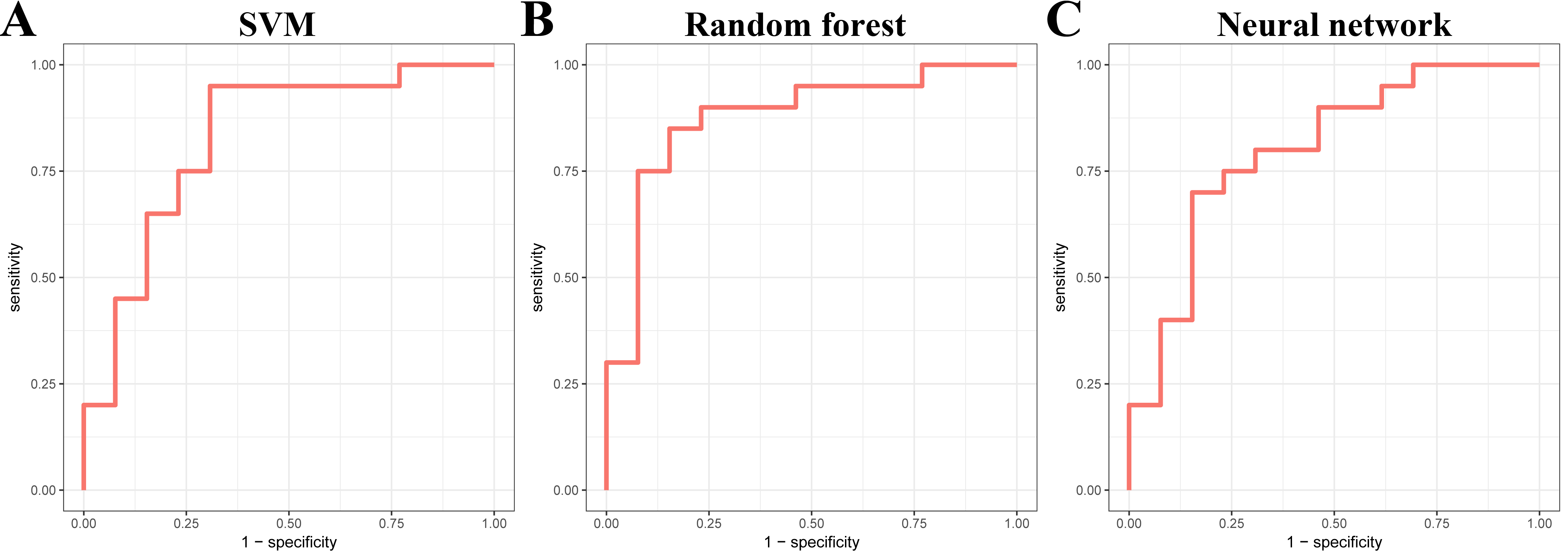

Supplement: Supplementary file 5 [file CAM4-9-1419-s005.tif]
