# Supplementary material for: FOLFOX treatment response prediction in metastatic or recurrent colorectal cancer patients via machine learning algorithms
Source: Cancer Med. 2020 Jan 1;9(4):1419–29. doi: 10.1002/cam4.2786 (PMC7013065; doi:10.1002/cam4.2786)
Supplement: Supplementary file 6 [file CAM4-9-1419-s006.doc]

| Table S1. The parameters of quality control for the included studies | | | | | | | |
| --- | --- | --- | --- | --- | --- | --- | --- |
| Datasets | IQC | EQC | CQCg | CQCp | AQCg | AQCp | Rank |
| GSE19860 | 4 | 3 | 0.04 | 0.28 | 0.04 | 0 | 2.5 |
| GSE28702 | 20 | 3 | 0.01 | 0.6 | 0.11 | 0.01 | 1.83 |
| GSE72970 | 2.67 | 3 | 0.22 | 6.49 | 0.06 | 0.03 | 1.67 |

IQC: internal quality control index; EQC: external quality control index; CQCg: consistency of differential expression quality control in genes; CQCp: consistency of differential expression quality control in pathways; AQCg: accuracy of differential expression quality control in genes; AQCp: accuracy of differential expression quality control in pathways.
