# Supplementary material for: FOLFOX treatment response prediction in metastatic or recurrent colorectal cancer patients via machine learning algorithms
Source: Cancer Med. 2020 Jan 1;9(4):1419–29. doi: 10.1002/cam4.2786 (PMC7013065; doi:10.1002/cam4.2786)
Supplement: Supplementary file 7 [file CAM4-9-1419-s007.doc]

| Table S2. Top 15 clusters with their representative enriched KEGG terms | | | | |
| --- | --- | --- | --- | --- |
| Term | Description | Log(*p*) | Log(q) | Symbols |
| hsa04140 | Autophagy - animal | -5.06 | -2.36 | AKT2, CTSB, ERN1, HRAS, PRKAA1, MAPK3, RPS6KB1, RRAS, BECN1, SNAP29, ATG14, ATG2A, WIPI2, SH3GLB1, ATG3, AKT1S1, ADCY3, SOD1, EIF4B, PRKCA, RPS6KA1, FZD3, LAMTOR1, STRADA, EGR1, SMAD3, PLCB3 |
| hsa04012 | ErbB signaling pathway | -3.13 | -0.94 | AKT2, AREG, HBEGF, EGFR, HRAS, PAK2, PRKCA, MAPK3, RPS6KB1, PAK4, ADCY3, ARF6, PLCB3, PTPN11, RRAS, DGKZ, PLPP3, AGPAT3, PCYT1A, CHPT1, FH |
| hsa05010 | Alzheimer's disease | -3.05 | -0.94 | ADAM10, ATP5F1D, CAPN1, COX4I1, COX5B, CYC1, ERN1, HSD17B10, NDUFA3, NDUFA10, NDUFB2, NDUFS3, PLCB3, MAPK3, NDUFS7 |
| hsa04137 | Mitophagy - animal | -2.76 | -0.94 | BNIP3L, HRAS, NBR1, RRAS, BECN1, SQSTM1, CALCOCO2, RHOT1 |
| hsa04144 | Endocytosis | -2.74 | -0.94 | BIN1, ARF6, AP2S1, EGFR, HRAS, LDLR, SMAD3, MDM2, RAB8A, TFRC, GIT2, STAM2, WASHC4, ARFGAP3, SH3GLB1, VPS28, SMURF2, RUFY1, ARAP1 |
| hsa05160 | Hepatitis C | -2.71 | -0.94 | AKT2, CLDN4, EGFR, HRAS, IRF3, IRF7, LDLR, OAS2, MAPK3, CLDN1, IRF9, OCLN |
| hsa01200 | Carbon metabolism | -2.71 | -0.94 | ACADM, ESD, FH, GOT2, MDH2, ALDH6A1, MTHFR, RPE, SUCLG1, RPIA, ACSS2, BCKDHA |
| hsa04068 | FoxO signaling pathway | -2.68 | -0.94 | AKT2, CCND2, CDKN2B, EGFR, HRAS, SMAD3, MDM2, PLK1, PRKAA, MAPK3, SGK2, NLK |
| hsa04210 | Apoptosis | -2.52 | -0.87 | AKT2, CAPN1, CTSB, ENDOG, ERN1, HRAS, LMNA, LMNB1, MAPK3, TUBA4A, PARP2, LMNB2 |
| hsa01523 | Antifolate resistance | -2.41 | -0.87 | FPGS, GART, ABCC1, MTHFR, SLC19A1 |
| M00391 | Exosome, eukaryotes | -2.39 | -0.87 | EXOSC7, EXOSC4, EXOSC5 |
| M00296 | BER complex | -2.39 | -0.87 | LIG3, PARP2, APEX2 |
| hsa05203 | Viral carcinogenesis | -2.37 | -0.87 | CCND2, CDC20, CDKN2B, ATF2, DDX3X, GTF2A1, HRAS, IL6ST, IRF3, IRF7, LTBR, MDM2, MAPK3, IRF9, VAC14 |
| hsa00100 | Steroid biosynthesis | -2.36 | -0.87 | DHCR7, LIPA, LSS, CYP2R1 |
| hsa04130 | SNARE interactions in vesicular transport | -2.23 | -0.81 | STX10, SNAP29, GOSR1, YKT6, USE1 |
