# Supplementary material for: FOLFOX treatment response prediction in metastatic or recurrent colorectal cancer patients via machine learning algorithms
Source: Cancer Med. 2020 Jan 1;9(4):1419–29. doi: 10.1002/cam4.2786 (PMC7013065; doi:10.1002/cam4.2786)
Supplement: Supplementary file 8 [file CAM4-9-1419-s008.doc]

| Table S3. 18 DEGs were identified via microarray meta-analysis and used in the prediction models | | | | | | | |
| --- | --- | --- | --- | --- | --- | --- | --- |
| Gene symbol | Protein | *p* | FDR | logFC | τ^2^ | Q | *P_Q_* |
| HELZ | Probable helicase with zinc finger domain | 0.00001 | 0.07667 | -0.75110 | 0 | 0.50645 | 0.77629 |
| PPP1R7 | Protein phosphatase 1 regulatory subunit 7 | 0.00027 | 0.24556 | 0.60412 | 0 | 0.90167 | 0.63710 |
| WASHC4 | WASH complex subunit 4 | 0.00030 | 0.24556 | -0.60110 | 0 | 1.09451 | 0.57854 |
| RNH1 | Ribonuclease inhibitor | 0.00035 | 0.24556 | 0.59378 | 0 | 0.87740 | 0.64487 |
| PKP3 | Plakophilin-3 | 0.00022 | 0.24556 | 0.61651 | 0 | 1.29726 | 0.52276 |
| LSS | Lanosterol synthase | 0.00028 | 0.24556 | 0.60248 | 0 | 0.14262 | 0.93117 |
| LYPLA2 | Acyl-protein thioesterase 2 | 0.00032 | 0.24556 | 0.59835 | 0 | 0.96696 | 0.61663 |
| DRAP1 | Dr1-associated corepressor | 0.00030 | 0.24556 | 0.59983 | 0 | 0.16321 | 0.92163 |
| IRF7 | Interferon regulatory factor 7 | 0.00024 | 0.24556 | 0.61022 | 0 | 0.07206 | 0.96461 |
| ERN1 | Serine/threonine-protein kinase/endoribonuclease IRE1 | 0.00023 | 0.24556 | -0.61273 | 0 | 0.47974 | 0.78673 |
| CCDC124 | Coiled-coil domain-containing protein 124 | 0.00022 | 0.24556 | 0.61392 | 0 | 0.02253 | 0.98880 |
| GCDH | Glutaryl-CoA dehydrogenase, mitochondrial | 0.00019 | 0.24556 | 0.62064 | 0 | 0.03530 | 0.98250 |
| RPS6KB1 | Ribosomal protein S6 kinase beta-1 | 0.00038 | 0.25000 | -0.58963 | 0 | 0.39555 | 0.82056 |
| C19ORF24 | Uncharacterized membrane protein C19orf24 | 0.00041 | 0.25238 | 0.58645 | 0 | 0.41196 | 0.81385 |
| APPBP2 | Amyloid protein-binding protein 2 | 0.00048 | 0.27022 | -0.65725 | 0.018958 | 2.35105 | 0.30866 |
| TSPAN17 | Tetraspanin-17 | 0.00053 | 0.28229 | 0.57472 | 0 | 0.18275 | 0.91267 |
| MLKL | Mixed lineage kinase domain-like protein | 0.00056 | 0.28275 | 0.57366 | 0 | 1.45882 | 0.48219 |
| EML3 | Echinoderm microtubule-associated protein-like 3 | 0.00063 | 0.29611 | 0.56749 | 0 | 0.56228 | 0.75492 |
