# Supplementary material for: FOLFOX treatment response prediction in metastatic or recurrent colorectal cancer patients via machine learning algorithms
Source: Cancer Med. 2020 Jan 1;9(4):1419–29. doi: 10.1002/cam4.2786 (PMC7013065; doi:10.1002/cam4.2786)
Supplement: Supplementary file 10 [file CAM4-9-1419-s010.doc]

| Table S5. Univariate and multivariate analyses of prognostic genes on overall survival for patients undergoing FOLFOX therapy | | | | | | | |
| --- | --- | --- | --- | --- | --- | --- | --- |
|  | Univariate | | |  | Multivariate | | |
|  | HR | 95% CI | *p* |  | HR | 95% CI | *p* |
| WASHC4 | 1.655 | 0.731-3.748 | 0.227 |  |  |  |  |
| HELZ | 1.152 | 0.652-2.037 | 0.626 |  |  |  |  |
| ERN1 | 1.143 | 0.664-1.966 | 0.629 |  |  |  |  |
| RPS6KB1 | 0.937 | 0.463-1.895 | 0.856 |  |  |  |  |
| APPBP2 | 1.518 | 0.860-2.679 | 0.150 |  |  |  |  |
| IRF7 | 0.647 | 0.392-1.067 | 0.088 |  |  |  |  |
| EML3 | 0.680 | 0.390-1.185 | 0.174 |  |  |  |  |
| LYPLA2 | 1.144 | 0.586-2.234 | 0.694 |  |  |  |  |
| DRAP1 | 0.755 | 0.379-1.505 | 0.425 |  |  |  |  |
| RNH1 | 0.928 | 0.502-1.717 | 0.812 |  |  |  |  |
| PKP3 | 0.564 | 0.325-0.979 | 0.042 |  | 0.982 | 0.499-1.931 | 0.957 |
| TSPAN17 | 0.603 | 0.309-1.177 | 0.138 |  |  |  |  |
| LSS | 0.495 | 0.297-0.824 | 0.007 |  | 0.690 | 0.387-1.230 | 0.208 |
| MLKL | 0.320 | 0.173-0.591 | <0.001 |  | 0.358 | 0.178-0.717 | 0.004 |
| PPP1R7 | 0.827 | 0.333-2.054 | 0.682 |  |  |  |  |
| GCDH | 0.533 | 0.242-1.174 | 0.118 |  |  |  |  |
| C19ORF24 | 0.581 | 0.347-0.975 | 0.040 |  | 0.918 | 0.480-1.754 | 0.795 |
| CCDC124 | 0.574 | 0.383-0.861 | 0.007 |  | 0.563 | 0.336-0.943 | 0.029 |

HR: hazard ratio; CI: confidence interval.
